# Histopathological Findings in Sleeve Gastrectomy Specimens: Prevalence, Determinants, and Clinical Implications in a Large Brazilian Cohort

**DOI:** 10.1007/s11695-026-08813-z

**Published:** 2026-06-30

**Authors:** Marina de Oliveira Menezes, Luiz Henrique Bandeira de Andrade Lima Filho, Luciana Teixeira de Siqueira, José Guido Corrêa de Araujo Júnior, Júlia Maria Mendes Lins, Álvaro Antônio Bandeira Ferraz

**Affiliations:** 1 Department of Surgery, Post-graduation in Surgery, Federal University of Pernambuco, Av. Prof. Moraes Rego, 1235 - Cidade Universitária, Recife CEP, 50670-901 Pernambuco, Brazil; 2 Department of Surgery, Federal University of Pernambuco, Recife Pernambuco, Brazil; 3 Medical School, Federal University of Pernambuco - UFPE, Recife Pernambuco, Brazil; 4 Medical School, University of Pernambuco - UPE, Recife Pernambuco, Brazil

**Keywords:** Obesity, Sleeve gastrectomy, Helicobacter pylori, Gastritis, Metaplasia, Bariatric surgery

## Abstract

**Background:**

The clinical relevance of routine histopathological examination of sleeve gastrectomy specimens remains controversial.

**Objective:**

To evaluate the prevalence of histopathological findings, their association with *Helicobacter pylori*, and potential implications for clinical management.

**Methods:**

Retrospective cross-sectional study including 1,475 patients undergoing sleeve gastrectomy (2014–2024) at a tertiary referral center in Brazil. Histopathological findings were classified according to the Updated Sydney System. Associations were analyzed using Poisson regression variance.

**Results:**

Inflammatory changes were observed in 55.1% of specimens, predominantly mild gastritis (73.2%). *H. pylori* was detected in 7.3%. Premalignant lesions were rare: atrophic gastritis (0.3%), intestinal metaplasia (0.6%), and one adenocarcinoma (0.07%). *H. pylori* was independently associated with gastritis (adjusted PR ranging from approximately 15 to 18, *p* < 0.001) and severity (*p* < 0.001). No association was observed with age or sex. Intestinal metaplasia was associated with age (*p* = 0.024).

**Conclusions:**

Histopathological abnormalities are common but predominantly mild. Clinically significant lesions are rare. Routine histopathological evaluation may detect unexpected findings in selected cases, but its universal role in risk stratification remains unclear.

## Key points

•A large post-screening cohort demonstrating low prevalence but non-zero risk of clinically relevant gastric pathology after sleeve gastrectomy.

•*Helicobacter pylori* infection was strongly associated with more severe and atrophic gastric changes, reinforcing its pathogenic role.

•Clinically relevant findings were detected despite preoperative screening, suggesting that important lesions may remain undiagnosed.

•Routine histopathological analysis may provide added value for risk stratification and should be considered in bariatric surgical practice.

## Introduction

Obesity is a chronic multifactorial disease and a major global public health problem [1, 2]. In addition to its well-known metabolic comorbidities, it is also associated with gastrointestinal changes and an increased risk of digestive tract neoplasms. Although lifestyle changes and drug therapies are part of the treatment, their long-term results are limited in cases of severe obesity. In this context, metabolic surgery stands out as the most effective strategy for sustained weight loss and improvement of associated comorbidities [3–8].

Obesity is associated with a range of gastrointestinal changes, including alterations in the intestinal microbiota, chronic low-grade inflammation, and hormonal changes driven by visceral fat accumulation. These factors contribute to a pro-inflammatory and potentially carcinogenic microenvironment [9, 10]. Indeed, epidemiological studies have consistently demonstrated an association between obesity and increased risk of several types of cancer, especially gastrointestinal tract neoplasms, including gastric cancer [11–16].

Among currently established surgical techniques, sleeve gastrectomy is the most commonly performed bariatric procedure worldwide, due to its relative technical simplicity, favorable safety profile, and significant weight-loss and mortality-reduction outcomes [17]. In addition to its restrictive effect, the procedure promotes important metabolic and hormonal changes, such as reduced ghrelin levels and modulation of adipokines, thereby contributing to improved glycemic control and insulin sensitivity, as well as improvements in conditions such as type 2 diabetes and hypertension [18, 19].

Despite the widespread use of sleeve gastrectomy, the role of routine histopathological analysis of surgical specimens remains controversial. Most specimens show nonspecific inflammatory changes, such as mild gastritis; however, potentially relevant findings, including Helicobacter pylori infection, atrophic gastritis, intestinal metaplasia, and, more rarely, neoplastic lesions, have been described. The prevalence of these changes varies widely across studies, and their association with patients’ clinical characteristics, such as sex and age, remains unclear [20–23]. Considering that H. pylori infection plays a central role in chronic gastric inflammation and gastric carcinogenesis, identifying these findings may have important clinical implications [22, 24–26].

Moreover, the absence of standardized recommendations regarding the need and clinical impact of routine histopathological evaluation, together with the methodological heterogeneity of available studies, keeps the debate open. Investigations with large samples and systematic evaluation of factors associated with gastric lesions, especially premalignant changes, are essential to clarify the relevance of these findings in the long-term follow-up of patients undergoing bariatric surgery.

This study aims to evaluate histopathological findings in a large cohort of sleeve gastrectomy specimens, analyze associations with *H. pylori* and demographic factors, and critically assess their clinical relevance.

## Methods

### Study Design and Ethical Aspects

Retrospective cross-sectional study of patients undergoing sleeve gastrectomy between 2014 and 2024 at two tertiary centers in Recife, Brazil, performed by the same surgical team.

Medical records of all patients who underwent laparoscopic sleeve gastrectomy between January 2014 and December 2024 were screened for eligibility (*n* = 1,641).

A total of 166 patients were excluded due to incomplete medical records including absence of histopathological reports of the resected gastric specimen.

The final study population consisted of 1,475 patients whose data were included in the analysis.

The study was conducted in accordance with the ethical principles of the 1964 Declaration of Helsinki and its later amendments, as well as the norms of the institutional ethics committee. This research project was approved by the Ethics Committee for Human Research of our institution, under the protocol CAAE 85742624.5.0000.8807.

### Data Collection and Variables

Demographic variables, including age and sex, were obtained from electronic medical records and analyzed for possible associations with the prevalence and severity of histopathological findings. Histopathological variables were extracted from the pathology reports of the resected gastric specimens. For standardization, the updated Sydney system was used to assess the presence and severity of gastritis (mild, moderate, and severe), as well as progression to atrophic gastritis and intestinal metaplasia. These were considered chronic forms of gastritis and classified as preneoplastic lesions [27]. This progression follows the currently accepted model of gastric carcinogenesis, the Correa cascade, which describes the slow, sequential evolution of histological changes that may culminate in gastric adenocarcinoma [28].

The presence of Helicobacter pylori and its association with other histopathological findings were systematically evaluated. H. pylori infection was identified by histological examination with Giemsa staining, as described in the pathology reports.

### Study Population

Eligible patients were those aged ≥ 16 years with a body mass index (BMI) ≥ 30 kg/m² who underwent sleeve gastrectomy during the study period and had a minimum postoperative follow-up of 12 months. Patients with incomplete records including the absence of a histopathological report of the surgical specimen were excluded.

### Primary Endpoint

- Prevalence of histopathological findings.

### Secondary Endpoints

- Association with *H. pylori*
- Association with demographic variables

### Statistical analysis

SPSS 31.0 (Statistical Package for the Social Sciences) for Windows, RStudio 2026.01.0, and Excel 365 were used for statistical analysis, with all tests conducted at a 5% significance level (p-value ≤ 0.05).

All results were calculated based on valid responses; ignored responses were not counted and are presented in tables with their respective absolute and relative frequencies. Numerical variables are represented by measures of central tendency and dispersion.

Associations between *Helicobacter pylori* infection and demographic and histopathological variables were initially assessed using chi-square or Fisher’s exact test, as appropriate. Crude and adjusted prevalence ratios (PR) with 95% confidence intervals were estimated using Poisson regression variance. Variables of epidemiological relevance and those associated with the outcome in bivariate analysis were considered for multivariable modeling. Due to the low frequency of atrophic gastritis and intestinal metaplasia, these variables were not included in the adjusted models.

The statistical analysis included:

- Descriptive statistics (mean, SD, proportions).
- Associations tested using:
  - ◦Poisson regression variance → prevalence ratios (PR)
- Multivariate models adjusted for:
  - ◦Age
  - ◦Sex
- Significance level: *p* < 0.05
- Software: RStudio 2026

## Results

A total of 1,475 histopathological exams of gastric specimens from patients undergoing sleeve gastrectomy between 2014 and 2024 were analyzed. The study population was predominantly female (76.3%), while 23.7% were male. Regarding age, 38.6% of patients were 30–39 years old, 37.6% were ≥ 40 years old, and 23.8% were < 30 years old, showing a balanced distribution between young and middle-aged adults.

Regarding anthropometric variables, the mean preoperative body mass index (BMI) was 40.1 ± 4.0 kg/m², with a median of 39.5 (IQR 37.2–42.2), ranging from 30.1 to 65.2 kg/m². After bariatric surgery, postoperative BMI decreased to a mean of 30.0 ± 4.6 kg/m² (median 29.4; IQR 26.7–32.7), with a range of 19.7–53.5 kg/m². Sociodemographic and anthropometric characteristics are presented in Table 1.

**Table 1:** Sociodemographic and anthropometric variables

Variables		*n*		%	
Sex					
Male		350		23.7	
Female		1125		76.3	
**Age**					
< 30		351		23.8	
30–39		570		38.6	
≥ 40		554		37.6	
	**Mean ± SD**	**Median (P25; P75)**		**Minimum–Maximum**	
Pre-op BMI	40.1 ± 4.0	39.5(37.2; 42.2)		30.1–65.2	
Post-op BMI	30.0 ± 4.6	29.4(26.7; 32.7)		19.7–53.5	

Histopathological analysis of gastric specimens revealed a high prevalence of inflammatory changes, observed in 55.1% of the specimens, while 44.9% were classified as normal gastric mucosa. Gastritis was the most frequent finding, identified in 54.8% of cases. Among these, mild gastritis was predominant (73.2%), followed by moderate (24.8%) and severe gastritis (2.0%). Premalignant lesions were infrequent in the sample. Atrophic gastritis was identified in 0.3% of cases, while intestinal metaplasia was present in 0.6% of samples. Helicobacter pylori infection was detected in 7.3% of the surgical specimens analyzed. The distribution of the main histopathological findings is described in Table 2.

**Table 2:** Types and prevalence of histopathological findings in resected stomachs after sleeve gastrectomy

Variables	*n*	%
Normal gastric mucosa		
Yes	662	44.9
No	813	55.1
Gastritis		
Yes	808	54.8
No	667	45.2
Gastritis severity		
Severe	16	2.0
Moderate	200	24.8
Mild	592	73.2
Atrophic gastritis		
Yes	4	0.3
No	1471	99.7
Metaplasia		
Yes	9	0.6
No	1466	99.4
H. pylori		
Yes	108	7.3
No	1367	92.7

A single case of gastric adenocarcinoma was identified in the entire sample. The patient was a 55-year-old woman who, on preoperative endoscopy, had been diagnosed with moderate chronic gastritis and tested negative for *H. pylori*. Histopathological examination of the resected specimen revealed adenocarcinoma, which was subsequently confirmed on postoperative endoscopy. Total gastrectomy was indicated; however, staging investigations performed both preoperatively and three months after sleeve gastrectomy demonstrated peritoneal carcinomatosis. The patient underwent drainage for chemotherapy but died one year later.

No statistically significant association was observed between age and H. pylori infection (*p* = 0.877). Compared with patients < 30 years old, the prevalence ratios (PRs) were 1.13 for individuals aged 30–39 years and 1.06 for those aged ≥ 40 years, with no significant difference between the groups. Similarly, there was no association between sex and H. pylori (*p* = 0.577), with a PR of 1.12 for males compared to females. On the other hand, gastritis showed a statistically significant association with H. pylori (*p* < 0.001). The presence of gastritis was associated with a PR of 17.01 (95% CI 6.97–41.48), indicating a higher prevalence of H. pylori compared to individuals without gastritis. The severity of gastritis also showed a significant association with infection (*p* < 0.001). Compared to mild gastritis, PRs were 4.80 for moderate and 6.00 for severe gastritis. A significant association was also observed between atrophic gastritis and H. pylori (*p* = 0.001), with a PR of 10.51 (95% CI 5.79–19.05). Conversely, intestinal metaplasia did not show a statistically significant association with H. pylori (*p* = 0.136), although a PR of 3.07 was observed, with a wide confidence interval (Table 3).

**Table 3:** Association between H. pylori, demographic data, and histopathological changes

Variables	H. pylori				
	Yes	No	PR	CI 95% PR	*p*-value
	*n* (%)	*n* (%)			
Age					0.877 *
< 30	24 (6.8)	327 (93.2)	1.00	---	
30–39	44 (7.7)	526 (92.3)	1.13	0.70; 1.82	
≥ 40	40 (7.2)	514 (92.8)	1.06	0.65; 1.72	
Sex					0.577 *
Male	28 (8.0)	322 (92.0)	1.12	0.74; 1.70	
Female	80 (7.1)	1045 (92.9)	1.00	---	
Gastritis					< 0.001 *
Yes	103 (12.7)	705 (87.3)	17.01	6.97; 41.48	
No	5 (0.7)	662 (99.3)	1.00	---	
Gastritis severity					< 0.001 *
Severe	6 (37.5)	10 (62.5)	6.00	2.96; 12.15	
Moderate	60 (30.0)	140 (70.0)	4.80	3.29; 7.00	
Mild	37 (6.3)	555 (93.8)	1.00	---	
Atrophic gastritis					0.001 **
Yes	3 (75.0)	1 (25.0)	10.51	5.79; 19.05	
No	105 (7.1)	1366 (92.9)	1.00	---	
Metaplasia					0.136 **
Yes	2 (22.2)	7 (77.8)	3.07	0.89; 10.58	
No	106 (7.2)	1360 (92.8)	1.00	---	

(*) Chi-square (**) Fisher’s exact test

In the crude analysis, *H. pylori* infection was not associated with age or sex. However, a strong association was observed between *H. pylori* and gastritis. Individuals with gastritis showed a markedly higher prevalence of *H. pylori* compared with those without gastritis. A dose-response pattern was also observed according to gastritis severity, with higher prevalence ratios among patients with moderate and severe gastritis. Atrophic gastritis and intestinal metaplasia were rare findings and, although descriptively associated with *H. pylori*, were not included in adjusted analysis due to sparse data (Tables 4 and 5).

**Table 4:** Association between *H. pylori* and demographic and histopathological variables: crude and adjusted prevalence ratios

Variables	Crude PR	95% CI	*p*-value
Age			
< 30	1.00	Reference	
30–39	1.13	0.70–1.82	0.877
≥ 40	1.06	0.65–1.72	
Sex			
Female	1.00	Reference	
Male	1.12	0.74–1.70	0.577
Gastritis			
No	1.00	Reference	
Yes	17.01	6.97–41.48	< 0.001

Crude and adjusted prevalence ratios were estimated using Poisson regression. Adjusted model included age, sex, and gastritis

**Table 5:** Association between *H. pylori* and demographic and gastritis severity variables: crude and adjusted prevalence ratios

Variables	Crude PR	95% CI	*p*-value
Age			
< 30	1.00	Reference	
30–39	1.13	0.70–1.82	
≥ 40	1.06	0.65–1.72	
**Sex**			
Female	1.00	Reference	
Male	1.12	0.74–1.70	
**Gastritis severity**			
Mild	1.00	Reference	
Moderate	4.80	3.29–7.00	< 0.001
Severe	6.00	2.96–12.15	< 0.001

The variable “gastritis” was not included in this model due to collinearity with gastritis severity

Atrophic gastritis did not show a statistically significant association with age (*p* = 0.372). Cases were rare and occurred mainly in the 30–39 and ≥ 40 years age groups, with no records in individuals < 30 years old. For intestinal metaplasia, a statistically significant association with age was observed (*p* = 0.024). Cases were identified in the < 30 and ≥ 40 years age groups, with no records in the 30–39 years group. No significant association was observed between gastritis and age (*p* = 0.881). The prevalence of the condition remained similar across the three age groups, at around 55%. Likewise, the severity of gastritis did not show a significant association with age (*p* = 0.425). In all age groups, mild gastritis was predominant, followed by moderate and severe forms (Table 6).

**Table 6:** Association between histopathological findings and age

Variables	Atrophic Gastritis					
	Yes	No		PR	CI 95% PR	*p*-value
	*n* (%)	*n* (%)				
Age						0.372 **
< 30	0 (0.0)	351 (100.0)		1.00	---	
30–39	1 (0.2)	569 (99.8)		***	***	
≥ 40	3 (0.5)	551 (99.5)		***	***	
	**Metaplasia**					
Age						0.024 **
< 30	4 (1.1)	347 (98.9)		***	***	
30–39	0 (0.0)	570 (100.0)		1.00	---	
≥ 40	5 (0.9)	549 (99.1)		***	***	
	**Gastritis**					
Age						0.881 *
< 30	195 (55.6)	156 (44.4)		1.03	0.91; 1.16	
30–39	314 (55.1)	256 (44.9)		1.02	0.92; 1.14	
≥ 40	299 (54.0)	255 (46.0)		1.00	---	
	**Gastritis severity**					
	**Severe**		**Moderate**		**Mild**	
	**n (%)**		**n (%)**		**n (%)**	
Age						0.425 *
< 30	5 (2.6)		53 (27.2)		137 (70.2)	
30–39	4 (1.3)		69 (22.0)		241 (76.7)	
≥ 40	7 (2.3)		78 (26.1)		214 (71.6)	

(*) Chi-square (**) Fisher’s Exact Test (***) Not calculable

## Discussion

This large cohort demonstrates that histopathological abnormalities are frequent after sleeve gastrectomy but predominantly mild. This result is consistent with previous studies demonstrating a high prevalence of chronic gastric inflammation in obese patients eligible for bariatric surgery, possibly related to the low-grade systemic inflammatory state characteristic of obesity, involving metabolic dysregulation, adipokine imbalance, and immune activation within the gastrointestinal tract [9, 10].

The prevalence of *H. pylori* (7.3%) is lower than expected for Brazil, likely reflecting preoperative screening and eradication strategies, selection bias in bariatric populations and/or limited sensitivity of histological detection.

Information on prior *H. pylori* diagnosis and eradication therapy was not systematically available, representing a major limitation and likely contributing to the low observed prevalence. However, all patients underwent upper digestive endoscopy pre-operatively. Even so, the presence of H. pylori was associated with several of the most clinically relevant histopathological findings, including atrophic gastritis, corroborating its central role in the pathophysiology of chronic gastric inflammation and the cascade of preneoplastic lesions [22]. The strong association between *H. pylori* and gastritis confirms its pathogenic role.

Premalignant lesions were rare (< 1%), consistent with previous studies. However, their detection, along with the single adenocarcinoma case, highlights the potential diagnostic value of histopathological examination. These changes may require specific endoscopic follow-up, H. pylori eradication when present, and long-term surveillance, especially in patients with additional risk factors. Thus, even in asymptomatic individuals and in the absence of significant changes on preoperative upper digestive endoscopy, histopathological examination of the surgical specimen may reveal conditions that could impact in clinical follow-up.

The absence of a statistically significant association between intestinal metaplasia and H. pylori infection probably reflects the small absolute number of cases, which limits the statistical power of the analyses. However, the coexistence of these findings in part of the sample reinforces the complexity of gastric carcinogenesis, which involves multiple environmental, infectious, and host-related factors. In addition, patients with more advanced histological changes tended to be older, although no strong statistical association was observed; this relationship is consistent with the temporal progression described in the cascade of inflammation–atrophy–metaplasia [27].

Our results contribute to the ongoing debate about the need for routine histopathological analysis of surgical specimens in sleeve gastrectomy, particularly in high-volume centers. Although the frequency of premalignant lesions was low, their detection has the potential to impact late clinical management and the oncological safety of these patients. This aspect becomes particularly relevant given the global increase in obesity and its association with gastrointestinal tract neoplasms.

Despite discussions regarding the cost-effectiveness of routine histopathological examination, our findings demonstrate that clinically relevant changes may be detected even in asymptomatic patients, supporting its role as an adjunctive risk-stratification tool. Nevertheless, these findings should be interpreted with caution, as the prevalence of pathology may be underestimated due to anatomically restricted sampling inherent to sleeve gastrectomy specimens.

Histopathological evaluation may provide incremental value in specific subgroups, particularly in patients with risk factors for gastric pathology, such as advanced age or evidence of *H. pylori* infection. Furthermore, the potential consequences of missed premalignant lesions, including the need for surveillance or eradication therapy, should be considered when weighing the benefits and costs of routine analysis.

This study has several limitations. Its retrospective design introduces potential information bias and limits the establishment of causal relationships. The lack of data regarding prior *H. pylori* infection and treatment is particularly relevant, as it may have influenced the observed prevalence and associated findings. Additionally, the low frequency of premalignant lesions limited the statistical power of some analyses. Histopathological evaluation is also inherently constrained by the partial nature of gastric resection in sleeve gastrectomy, potentially leading to underestimation of focal lesions in non-resected areas, particularly the gastric antrum. Nevertheless, although the majority of the specimen in vertical sleeve gastrectomy comprises the gastric body and fundus, a portion of the antrum is typically included, partially mitigating this limitation.

Despite these limitations, this study has notable strengths, including a large sample size, an extended study period, and standardized histopathological assessment. These features enhance the robustness and external validity of the findings, particularly for comparable bariatric populations in Latin American settings.

## Conclusion

In this large cohort, histopathological alterations were common but predominantly mild, whereas premalignant lesions and *Helicobacter pylori* infection were infrequent. Although routine histopathological examination may occasionally identify unexpected clinically relevant findings, its role as a universal risk-stratification tool remains unclear. Prospective studies integrating detailed preoperative endoscopic assessment with longitudinal clinical outcomes are warranted to better define its clinical utility and cost-effectiveness.

## Data Availability

No datasets were generated or analysed during the current study.
